# Paraclitoral Epidermal Cysts: A Literature Systematic Review

**DOI:** 10.3390/medicina61030520

**Published:** 2025-03-17

**Authors:** Clarissa Costa, Marta Barba, Alice Cola, Matteo Frigerio

**Affiliations:** 1 Department of Gynecology and Obstetrics, University of Milano-Bicocca, 20126 Monza, Italy; c.costa14@campus.unimib.it; 2 Fondazione IRCCS San Gerardo dei Tintori, 20900 Monza, Italy; m.barba8792@gmail.com (M.B.); alice.cola1@gmail.com (A.C.)

**Keywords:** clitoral cyst, epidermoid cyst, epidermal inclusion cyst, inclusion cyst, spontaneous cyst of clitoris

## Abstract

*Background and Objectives*: Epidermal inclusion cysts are benign lesions with usually good prognosis. Clitoral epidermal cysts are very uncommon and can be spontaneous or caused by a prior surgical operation, usually female genital mutilation. Surgical removal of the cyst is the preferred treatment, and it is associated with excellent postoperative results. *Materials and Methods*: We present the medical history and surgical treatment of a 39-year-old woman with a symptomatic epidermal clitoral cyst. To discuss our case in the context of a synopsis of similar published cases, we conducted a systematic review of epidermal clitoral cysts to synthesize available knowledge on symptoms, diagnosis, etiology, and management about this rare condition. *Results*: A total of 46 studies, describing 162 patients, were identified. The presence of clitoral cysts should be suspected in patients complaining of vaginal bulging, sexual discomfort, and urinary symptoms. Clinical examination as well as other instrumental techniques represent a useful tool for diagnosis. The gold standard treatment is to perform surgical excision. Follow-up is needed to diagnose possible recurrences. *Conclusions*: We present the first systematic review focusing on epidermal clitoral cysts including both spontaneous and traumatic etiologies. It is important to include these lesions in the differential diagnosis of congenital clitoromegaly and clitoral mass, especially in the case of history of FGM, trauma, or surgery in the genital area.

## 1. Introduction

Epidermal inclusion cysts are benign lesions with a generally favorable prognosis that can be found on various parts of the body, such as the back, chest, neck, face, scalp, and less commonly, the vulva [1]. Epidermoid cysts are typically solitary, round, asymptomatic, and slow-growing proliferations of epidermal cells containing keratinous debris, caused by the abnormal proliferation of epidermal cells [2]. Clitoral epidermoid cysts may occur spontaneously or result from previous surgical procedures [3], with a significant proportion being linked to female genital mutilation (FGM), a practice that remains prevalent in certain cultural contexts [4].

Although epidermoid cysts are typically asymptomatic and have a favorable prognosis, vascular complications can arise, particularly when the cysts are not fully excised or rupture. The delicate vascular anatomy of the clitoral region makes it critical to approach surgical excision with precision to avoid damaging these structures. Inadequate removal of the cyst or inadvertent trauma to the vascular supply during surgery can result in hemorrhage, delayed healing, and potentially long-term vascular compromise. Such complications could impair sexual function, causing discomfort, reduced sensitivity, and other related issues [5]. Additionally, if a cyst ruptures, keratinous material can spill into surrounding tissues, leading to inflammatory responses, localized cellulitis, and abscess formation. Infection is another potential complication, often exacerbated by incomplete excision or poor post-surgical care. The surrounding vascular tissue may become inflamed or infected, leading to erythema, swelling, and pain, all of which can further complicate recovery. Lymphatic involvement may also occur, potentially causing lymphangitis or lymphadenopathy, particularly if the cyst is large or if it has been present for an extended period [6].

Differential diagnoses include congenital anomalies, hormonal imbalances, neoplastic growths, inflammatory, or infectious lesions, which may contribute to delayed diagnosis and treatment. Diagnosis is primarily clinical, based on the cystic appearance, although radiologic imaging such as pelvic ultrasound and magnetic resonance imaging (MRI) can help confirm the diagnosis [1]. Laboratory tests may also be helpful to rule out hormonal causes of clitoromegaly.

While surgical excision remains the gold standard for treating epidermal inclusion cysts, alternative, less invasive approaches may be considered in certain cases, particularly for small, non-infected cysts or for patients who are not ideal surgical candidates. These include aspiration or drainage, intralesional steroid injections to reduce inflammation [7], carbon dioxide (CO_2_) laser or radiofrequency ablation [8], and topical or systemic antibiotics.

We present a case of a large clitoral epidermal inclusion cyst and review the literature on similar cases.

## 2. Case Description

A 39-year-old Egyptian woman was referred to our urogynecology division due to vaginal bulging symptoms, without urinary symptoms or dyspareunia. There was no history of female circumcision, no family history of inborn defects, and no genital abnormalities in her medical record. On clinical examination, a 6–7 cm paraclitoral mass was noted, which caused deep anatomical subversion, with an apparent cleavage plane observed during ultrasound examination. Following both clinical and anamnestical evaluation, with no evidence of malignant characteristics or endocrinological causes, a diagnosis of paraclitoral cyst was made. After proper counseling, the patient was scheduled for paraclitoral cyst excision. She received broad-spectrum intravenous antibiotic prophylaxis (cefazolin).

The patient provided written informed consent for the publication and the use of her images and video.

The surgical procedure was carried out as follows (Video 1):

1. A longitudinal cutaneous incision was performed at the interlabial sulcus.
2. Gentle dissection was performed until the cystic wall was identified.
3. Dissection was carried on until the base of the lesion was reached at the junction between the clitoral body and the right crura.
4. The cyst was then totally excised while preserving the clitoris.
5. Primary layered repair was performed to ensure proper clitoral reconstruction.
6. Excess tissue was trimmed, and the cutaneous incision was sutured to complete the procedure.

**Figure 1:**
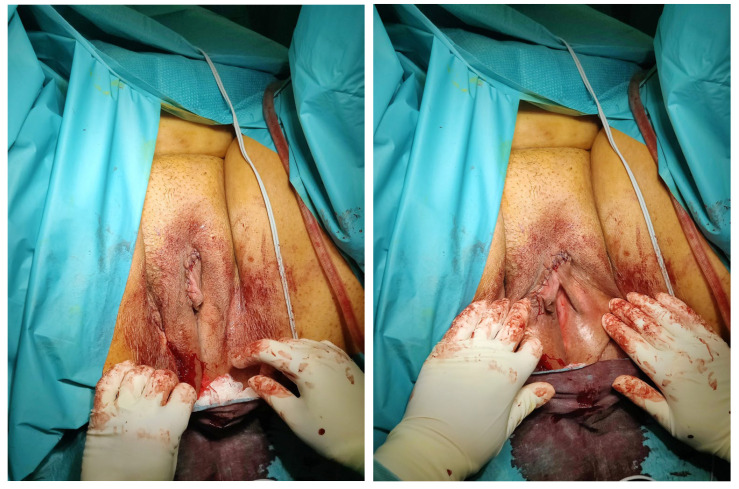
Postoperative outcome.

The procedure was completed in 63 min, with blood loss less than 100 mL. No surgical complications were observed. The patient was successfully discharged on postoperative day 1. The definitive histopathological examination confirmed the diagnosis of a clitoral epidermal cyst. At the current follow-up, the patient remains asymptomatic (Figure 1).

## 3. Systematic Review of Epidermal Clitoral Cysts

### 3.1. Patients and Methods

A systematic search of studies was conducted across four databases (PubMed, Scopus, ISI Web of Science, and Science Direct) using a combination of keywords and text terms, including “clitoral cyst”, “clitoral epidermal cyst”, and “epidermoid cyst”. Two reviewers (C.C. and M.B.) independently screened the titles and abstracts of the records retrieved through the database searches.

Only papers published after 2000 and in English were considered. Additionally, a manual search was performed to include relevant articles by reviewing the reference lists of key studies. Full texts of records recommended by at least one reviewer were independently screened by the same two reviewers and assessed for inclusion in the systematic review. Disagreements between reviewers were resolved by consensus.

The systematic review was conducted and reported in accordance with the PRISMA Statement for Reporting Systematic Reviews and Meta-Analyses. Data selection and extraction were based on the PICOS framework (Population, Intervention, Comparison, Outcome, Study Type) using a piloted form specifically designed to capture information on study characteristics. Data were extracted independently by two authors to ensure accuracy and consistency.

### 3.2. Results

**Figure 2:**
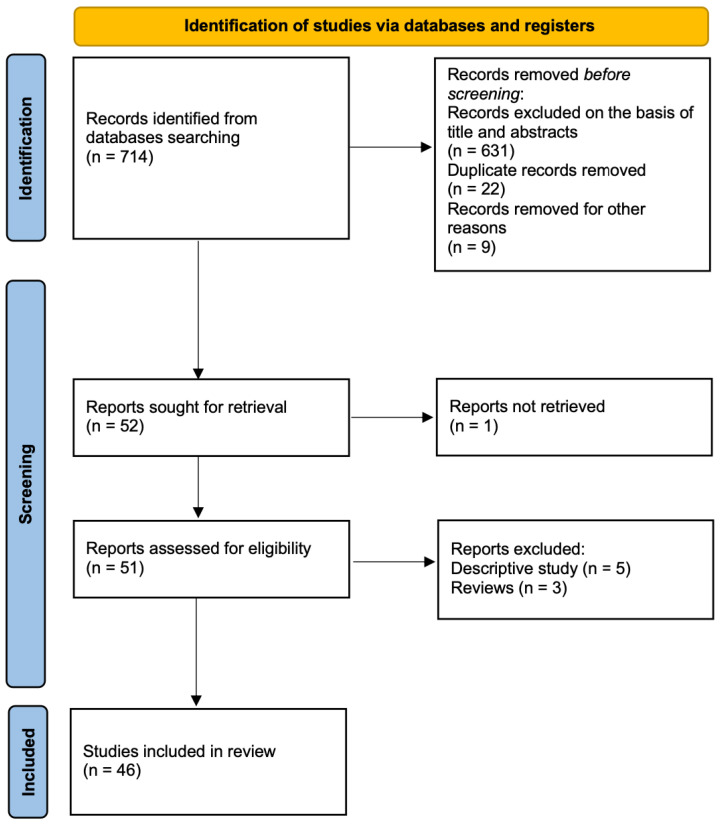
PRISMA flow chart.

The electronic database search provided a total of 714 results (Figure 2). Of them, 631 were not relevant to the review based on title and abstract screening, and 9 were excluded for being in languages other than English. After duplicate exclusion, 52 citations were left. After deleting reviews, descriptive articles, and articles without published full texts, 46 articles were finally included. All the included studies were case reports and retrospective case series published from January 2000 to January 2024, describing a total of 162 patients. The main characteristics of these studies are listed in Table 1.

**Table 1:** Main characteristics of the studies included in the systematic review. MRI = magnetic resonance imaging; FGM = female genital mutilation; CT = computed tomography.

Ref	First Author	Year	Country	N	Age (years)	Symptoms	Physical Exam	Etiology	Instrumental Diagnostic	Size (cm)	Management	Complications/Recurrence
[1]	DiCarlo-Meacham	2020	USA	1	15	Loss of clitoral sensation, anorgasmia	Hypoestrogenic-appearing vulvar epithelium, mass located superior to the urethra, inability to retract the clitoral hood to visualize the clitoris	Spontaneous	Pelvic MRI	4	Surgical excision	No
[2]	Anderson-Mueller	2009	USA	1	17	Clitoral pain and clitoromegaly	Enlarged clitoris with a possibly cystic area on the end	Spontaneous	Pelvic MRI	6.5	Surgical excision	No
[3]	Rouzi	2010	Saudi Arabia	31	±28	Clitoral mass	Clitoral cyst	FGM	/	±4	28: Surgical excision 3: others	No
[4]	Rouzi	2001	Saudi Arabia	21	±18	21: Clitoral mass 1: dysuria 1: dyspareunia	Clitoral cyst	FGM	/	±4	Surgical excision	No
[5]	Wu	2016	USA	1	27	Clitoral mass, mild erythema, limited clitoral stimulation and discomfort	Distention of the clitoral hood, fusion of the superior labia majora and minora	Previous genital trauma	/	3	Surgical excision	No
[9]	Oluwaferanmi	2022	UK	1	31	Clitoral mass	8 cm, soft and fluctuant, mildly tender clitoral swelling	Spontaneous	Pelvic MRI	8	Surgical excision	No
[10]	Ibrahimi	2021	Morocco	1	33	Sexual discomfort, difficulty in urination, clitoral mass	Painless midline mobile cystic mass	Spontaneous	Ultrasound	5	Surgical excision	No
[11]	Huebner	2024	USA	1	5	Clitoromegaly and worsening vulvovaginitis with itching and burning	Mobile, soft, peri-clitoral mass and surrounding hypopigmentation consistent with LS	Lichen Sclerosus	Ultrasound	/	Surgical excision	Small drainage 1 week post-operation
[12]	Zoorob	2019	USA	1	39	Tender periclitoral fluctuant lesion	Dyspareunia, periclitoral swelling and pain	FGM	/	4	Surgical excision	No
[13]	Fux-Otta	2022	Argentina	1	16	Clitoral mass	Painless clitoral mass	Spontaneous	Ultrasound	4	Surgical excision	No
[14]	Shober	2014	USA	1	5	Urinary spraying and infrequent volitional voiding	Slightly prominent clitoral hood	Spontaneous	Ultrasound	2.5	Surgical excision	No
[15]	Prasad	2022	India	1	43	Swelling in the perineal region, discomfort and dyspareunia	Well-circumscribed, mobile, round, soft, cystic, fluctuant, mildly tender swelling of the clitoral area	Spontaneous	/	6	Surgical excision	No
[16]	Beurdeley	2011	France	1	11	Clitoral mass	Rounded, mobile soft mass at the clitoral region	Spontaneous	Pelvic MRI, ultrasound	5	Surgical excision	No
[17]	Hughes	2013	USA	1	32	Painless clitoral enlargement	Enlarged clitoris with a palpable soft fluctuance at the base	Spontaneous	Pelvic MRI, ultrasound	6	Surgical excision	No
[18]	Karaci	2019	Turkey	1	22	Clitoral mass	Mobile, soft, non-fluctuant clitoral mass	FGM	Pelvic MRI	6	Surgical excision	No
[19]	Ozturk	2018	Turkey	1	36	Enlarged and painful clitoral mass with vitiligo lesions, sexual difficulties and psychological distress	Mobile, non-tender, rounded cystic swelling in the clitoral area	FGM	/	13	Surgical excision	No
[20]	Aggarwal	2010	India	1	5	Clitoral mass	Clitoral enlargement with cystic mass	Spontaneous	Ultrasound	4	Surgical excision	No
[21]	Asante	2010	USA	1	37	Discomfort with walking, dyspareunia	Mobile, non-tender, rounded cystic swelling in the right periclitoral area	FGM	/	4	Surgical excision	No
[22]	Paulus	2010	USA	1	47	Clitoral mass and pain, dyspareunia, pain with ambulation	Non-tender, non-erythematous, compressible mass	Spontaneous	/	20	Surgical excision	No
[23]	Beltrão	2019	Brasile	1	4	Asymptomatic	Clitoral enlargement with cystic mass	Spontaneous	Ultrasound	2	Conservative treatment and spontaneous regression	/
[24]	Victoria-Martínez	2016	Spain	1	39	Painful genital swelling, psychological discomfort	Clitoral nodular lesion covered with preserved mucosa	FGM	/	3	Surgical excision	No
[25]	Birge	2019	Turkey	1	43	Dyspareunia	Well-circumscribed, mobile, non-tender, rounded, cystic swelling in the right periclitoral area	FGM	Ultrasound	10	Surgical excision	No
[26]	Yoong	2004	UK	1	29	Dyspareunia, pain with ambulation	Midline fluctuant paraclitoral cyst	FGM	/	8	Surgical excision	No
[27]	Celik	2011	Turkey	1	9	Clitoral mass	Soft, mobile, non-tender, rounded clitoral swelling	Previous genital trauma	/	3	Surgical excision	No
[28]	Lambert	2011	Canada	1	23	Asymptomatic	Clitoral cyst	Spontaneous	/	3	Surgical excision	No
[29]	Mahmoudnejad	2020	Iran	3	1:45 2:35 3:25	1: Progressively painless enlarging clitoral mass 2: Non-tender clitoral mass, difficulty in voiding and dyspareunia 3: Gradual swelling of the clitoral region, urinary frequency, and difficult voiding	1: Mobile, non-tender, soft, well defined clitoral mass 2: Mobile, fluctuant, and firm cystic lesion at the clitoral site 3: Round and slightly tender clitoral mass with surface ecchymosis	Spontaneous	1, 2, 3: /	1:5 2:3 3:5	1, 2, 3: Surgical excision	1, 2, 3: No
[30]	Al-Ojaimi	2013	United Arab Emirates	1	19	Enlarged and painful clitoral mass, sexual difficulties, and psychological distress	Large, soft, mobile, non-tender, fluctuant cyst at the site of the clitoris	Spontaneous	Ultrasound	8	Surgical excision	No
[31]	Rizk	2009	United Arab Emirates	2	1:30 2:47	1: Painless clitoral mass 2: Painless clitoral mass, difficulties in having sexual intercourse	1,2: rounded, cystic, mobile, non-tender and compressible clitoral swelling	FGM	1, 2: /	1:6 2:9	1, 2: surgical excision	1, 2: no
[32]	Johnson	2012	USA	1	15	Clitoral mass	Clitoral mass arising from the superior aspect of the gland and covered by the clitoral hood	Spontaneous	Pelvic MRI	5	Surgical excision	No
[33]	Cetinkurşun	2009	Turkey	1	1.5	Clitoromegaly	Rounded, cystic, mobile, and non-tender mass at the clitoral region	Spontaneous	Ultrasound	3	Surgical excision	No
[34]	Saha	2013	India	1	5	Painless swelling at external genitalia	Firm cystic, non-tender, mobile swelling arising from the clitoris	Spontaneous	Ultrasound	3	Surgical excision	No
[35]	Fedele	2008	Italy	1	22	Clitoromegaly	Median, mobile, and soft clitoral mass	Spontaneous	Pelvic MRI, ultrasound	7	Surgical excision	No
[36]	Linck	2001	USA	1	28	Clitoromegaly	Severe, soft, mobile, and non-fixed clitoral enlargement	Spontaneous	Intravenous pyelogram, ultrasound	12	Surgical excision	No
[37]	Kroll	2000	USA	1	19	Clitoral mass, mild pruritus and tenderness, pain with ambulation	Fluctuant superficial and mobile mass arising at the site of the previous clitoral excision	FGM	/	8	Surgical excision	No
[38]	Okwudili	2011	Nigeria	1	18	Low level of painless clitoral swelling, gradually increasing in size to occlude the whole introitus	Left cystic mass involving the left labia majora and overlying the introitus	FGM	Ultrasound	10	Surgical excision	No
[39]	El-agwany	2013	Egypt	1	40	Vulvar swelling causing dragging pain during standing and walking, dyspareunia and splashing of urine stream on micturition with dysuria	Anterior midline cystic mass at site of the circumcision scar and occluding the introitus, non-tender, no warm, soft in consistency and fluctuant	FGM	Ultrasound	15	Surgical excision	No
[40]	Aimen	2016	France	1	36	Clitoral mass	Vulvar tumor covered by a regular skin with a soft consistency and no adhesion to the deep plane	FGM	Ultrasound	7	Surgical excision	No
[41]	Apostolis	2012	USA	1	21	Slowly enlarging periclitoral mass, occasional dyspareunia, chronic constipation and occasional dyschezia	Irregular, indurated, and erythematous left labia majora	Spontaneous	CT scan	10	Surgical excision and hematoma drainage	No
[42]	Doan	2021	Switzerland	1	0	Clitoromegaly	Clitoral enlargement with a solid, rounded, tense and erythematous mass	Spontaneous	Pelvic MRI, ultrasound	2	Surgical drainage	No
[43]	Dun	2016	USA	1	30	Non-tender, midline clitoral mass	Non-tender, cystic mass located in the midline above the urethra	FGM	Pelvic MRI	8	Surgical excision	No
[44]	Yang	2011	Taiwan	1	33	Right painless vulvar mass	Soft, movable, and non-tender mass in the right labia majora extended to the pubic rami anteriorly, buttock posteriorly, inner thigh laterally, and labia minora and clitoris medially	Spontaneous	Pelvic MRI, ultrasound	15	Surgical excision	No
[45]	Gomes	2013	Brazil	1	2	Clitoromegaly	Cyst formation on the clitoral body, with well-defined limits and no adherence to deeper planes	Spontaneous	/	/	Surgical excision	No
[46]	Osifo	2010	Nigeria	37	±17	Clitoromegaly, dragging vulvar sensation and bulging, psychological discomfort, sexual difficulties, pain with deambulation	Clitoral cyst	FGM	/	±7	Surgical excision	No
[47]	Diouf	2017	Dakar	8	±23	Aesthetic discomfort, clitoral cyst	Rounded painless mass that is non-pulsatile, sessile and movable superficially and in depth	FGM	/	±11.5	Surgical excision	No
[48]	Davari	2006	Iran	1	24	Painful clitoral mass	Cystic mass extended from the clitoral region into the labia minora	Spontaneous	Cystoscopy	12	Surgical excision	No
[49]	Şahin	2023	Turkey	21	±33	Discomfort mass in the vagina	Clitoral cyst	FGM	/	5	Surgical excision	No

Patient age ranged from 0 to 47 years with a median of 24 years and a mean of 23.6 ± 13.1 years. The chief complaint was the presence of clitoral mass and clitoromegaly (*n =* 155). Other symptoms included sexual dysfunctions or discomfort (*n =* 52), urological symptoms (*n =* 6), pain with deambulation (*n =* 42), and psychological distress (*n =* 40). Only two patients were asymptomatic. In total, 132 cases were associated with female genital mutilation, 27 cases were spontaneous cysts, 2 cases were associated with genital trauma, and 1 case was the initial presentation of lichen sclerosus. On physical examination, all the patients presented a well-circumscribed clitoral or paraclitoral cyst, with an average cyst dimension of 5.9 cm. In 27 patients, an instrumental diagnostic was performed, with ultrasound examination being the most used to confirm the clitoral cyst. It was performed alone in 13 cases, in combination with magnetic resonance imaging (MRI) in 5 patients, and with intravenous pyelogram in 1 case. In six cases, MRI was the only diagnostic tool used, while in two cases, other techniques were used (CT scan and cystoscopy). Almost all patients received surgical therapy (*n =* 161) and only one patient underwent follow-up and subsequent spontaneous cyst regression. Among surgical procedures, surgical excision was performed alone in 156 patients and in combination with hematoma drainage in 1 patient. Other techniques included surgical drainage and other surgical techniques (*n =* 4). Post-operative complications occurred in only one case and no recurrence was observed at the post-operative follow-up.

### 3.3. Discussion

Our analysis highlights the clinical presentation, diagnostic approaches, and management of clitoral cysts in a diverse patient population. The age distribution ranged from infancy to adulthood, suggesting that these cysts can develop at various life stages, though they are more commonly observed in pre-menopausal women. The predominant complaint among patients was the presence of a clitoral mass and clitoromegaly, which often led to associated symptoms causing a significant impact on both physical and psychological well-being.

Clitoral enlargement can result from both congenital malformations and acquired conditions. Congenital forms are typically noticeable at birth, whereas acquired forms can develop at any age. The most common hormonal causes of congenital clitoromegaly include congenital adrenal hyperplasia or other forms of androgen excess in females. Depending on the severity, closure of the labio-scrotal folds and clitoral enlargement may be evident at birth. In cases of severe congenital clitoromegaly, clitoroplasty with preservation of sensitivity is the preferred treatment [50].

Acquired forms of clitoromegaly can be either hormonal or non-hormonal. Among the hormonal causes, hyperandrogenism and hypertestosteronism due to endocrinopathies or hormonally active masculinizing tumors are most frequently cited. The primary endocrinopathies are non-polycystic ovarian hypertestosteronism and polycystic ovarian syndrome. The only reported non-hormonal cause is neurofibromatosis [51]. Neurofibromatosis affecting the genitourinary tract is exceedingly rare, with clitoral involvement being the most commonly reported [52]. In these cases, clitoromegaly tends to be irregular in shape, asymmetrical, and rough in appearance [53]. Bladder involvement is frequent, potentially leading to outflow obstruction or poor vesical compliance [54]. Various cysts may also form in the clitoral region, causing significant clitoromegaly. Congenital cystic lesions in the vaginal area have a prevalence of around 0.6% [22]. These conditions are often mistaken for ambiguous genitalia when present at birth but typically resolve spontaneously without intervention.

Although clitoral epidermal inclusion cysts can have a spontaneous origin, they are most commonly associated with female genital mutilation (FGM), as shown in our systematic review.

It is estimated that 101 million girls aged 10 and older in Africa have undergone some form of FGM, leading to an increase in reports of epidermal inclusion cysts and other related complications [14]. FGM is classified into four major types: type I (clitoridectomy), which involves the total or partial removal of the clitoris and, rarely, the prepuce; type II (excision), which involves the total or partial removal of the clitoris and labia minora, with or without excision of the labia majora; type III (infibulation), the most severe form, involving the total or partial removal of the labia minora and majora, with or without clitoridectomy; and type IV, which encompasses all other harmful procedures to the female genitalia for non-medical reasons [21]. Long-term consequences of FGM include obstetrical, gynecological, and psychological issues, such as dyspareunia, menstrual disorders, chronic vaginal and pelvic infections, recurrent urinary tract infections, infertility, depression, low libido, and anxiety. It can also increase the risk of complications during pregnancy, childbirth, and infant mortality [55]. These conditions can significantly impair quality of life and require tailored management. Given the profound impact of FGM on women’s physical and mental well-being, identifying and addressing these complications early through clinical interventions is critical.

Clinically, the diagnosis of a clitoral epidermoid cyst is often straightforward based on patient history and physical examination. A history of FGM, trauma, or previous genital surgery can serve as crucial clues in identifying the condition. In cases where cysts are symptomatic, particularly those causing sexual dysfunction, urological issues, or psychological distress, intervention is necessary.

Ultrasound and MRI are the most commonly used imaging modalities, providing important information on cyst size, location, and relationship to surrounding structures. These tools are indispensable in the preoperative assessment of patients with clitoral cysts, especially in differentiating benign cysts from other pathological conditions.

Surgical treatment is typically required for clitoral epidermoid cysts, with excision being the gold standard. However, the surgical approach poses challenges, particularly in preserving clitoral sensitivity and avoiding damage to surrounding structures, which are essential for sexual function. In cases of cysts associated with FGM, careful dissection is necessary to remove the cyst while preserving the clitoral tissue and minimizing scarring. Postoperative complications, while rare, can include hematoma formation, infection, and recurrence. However, the majority of patients do not experience recurrence or significant complications following surgical excision. It is important to counsel patients on potential outcomes, including the risk of sexual dysfunction or sensitivity changes post-surgery [30].

To the best of our knowledge, this is the first systematic review focused on epidermal clitoral cysts, including both spontaneous and traumatic causes. The limitations of this study include a limited sample size due to the rarity of the condition, potential selection bias caused by restricting the analysis to English-language publications, and the inclusion of studies published only from the year 2000 onward. Additionally, this review is primarily based on isolated case reports, which may introduce publication bias. Nevertheless, we were able to describe the symptoms, etiology, management, and complications of clitoral epidermoid cysts as found in the literature.

## 4. Conclusions

Despite being a rare condition, clitoral epidermoid cysts should be considered in the differential diagnosis of clitoromegaly and clitoral masses. Prompt surgical intervention is essential for managing symptoms and preventing complications. This review highlights the importance of early recognition, appropriate surgical management, and the need for comprehensive patient care in improving clinical outcomes.

## Data Availability

No new data were created or analyzed in this study.
